# Time interval between the diagnosis of breast cancer and brain metastases impacts prognosis after metastasis surgery

**DOI:** 10.1007/s11060-022-04043-2

**Published:** 2022-06-07

**Authors:** Anna Michel, Thiemo Florin Dinger, Alejandro N. Santos, Daniela Pierscianek, Marvin Darkwah Oppong, Yahya Ahmadipour, Philipp Dammann, Karsten H. Wrede, Jörg Hense, Christoph Pöttgen, Antonella Iannaccone, Rainer Kimmig, Ulrich Sure, Ramazan Jabbarli

**Affiliations:** 1 Department of Neurosurgery and Spine Surgery, University Hospital Essen, Essen, Germany; 2 Department of Medical Oncology, University Hospital Essen, Essen, Germany; 3 Department of Radiotherapy, University Hospital Essen, Essen, Germany; 4 Department of Obstetrics and Gynecology, University Hospital Essen, Essen, Germany; 5 Center for Translational Neuro- & Behavioral Sciences (C-TNBS), University Duisburg Essen, Essen, Germany; 6 German Cancer Consortium (DKTK) Partner Site, University Hospital Essen, 45147 Essen, Germany; 7 Department of Neurosurgery and Spine Surgery, University Hospital Essen, University Duisburg-Essen, Hufelandstraße 55, 45147 Essen, Germany

**Keywords:** Breast cancer, Brain metastasis, Time interval, Brain metastasis surgery, Invasive loboluar breast cancer

## Abstract

**Purpose:**

Breast cancer (BC) is the most frequently diagnosed tumor entity in women. Occurring at different time intervals (TI) after BC diagnosis, brain metastases (BM) are associated with poor prognosis. We aimed to identify the risk factors related to and the clinical impact of timing on overall survival (OS) after BM surgery.

**Methods:**

We included 93 female patients who underwent BC BM surgery in our institution (2008–2019). Various clinical, radiographic, and histopathologic markers were analyzed with respect to TI and OS.

**Results:**

The median TI was 45.0 months (range: 9–334.0 months). Fifteen individuals (16.1%) showed late occurrence of BM (TI ≥ 10 years), which was independently related to invasive lobular BC [adjusted odds ratio (aOR) 9.49, 95% confidence interval (CI) 1.47–61.39, p = 0.018] and adjuvant breast radiation (aOR 0.12, 95% CI 0.02–0.67, p = 0.016). Shorter TI (< 5 years, aOR 4.28, 95% CI 1.46–12.53, p = 0.008) was independently associated with postoperative survival and independently associated with the Union for International Cancer Control stage (UICC) III–IV of BC (aOR 4.82, 95% CI 1.10–21.17, p = 0.037), midline brain shift in preoperative imaging (aOR10.35, 95% CI 1.09–98.33, p = 0.042) and identic estrogen receptor status in BM (aOR 4.56, 95% CI 1.35–15.40, p = 0.015).

**Conclusions:**

Several factors seem to influence the period between BC and BM. Occurrence of BM within five years is independently associated with poorer prognosis after BM surgery. Patients with invasive lobular BC and without adjuvant breast radiation are more likely to develop BM after a long progression-free survival necessitating more prolonged cancer aftercare of these individuals.

**Supplementary Information:**

The online version contains supplementary material available at 10.1007/s11060-022-04043-2.

## Introduction

Breast cancer (BC) is one of the most frequent cancer entities in women with increasing rates of brain metastasis (BM). [1, 2] Multimodal treatment concepts for BC include the surgical and (neo-) adjuvant options with radiation, conventional chemotherapy, endocrine therapy, target therapy like anti-human epidermal growth factor 2 (HER2), as well as the treatment of distant metastases. [3]

The prevalence of BM varies between 15 and 50% depending on the presence of different tumor features. [4–6] Along with synchronous occurrence, BM might also develop in the further course of disease. The time interval (TI) for the occurrence of BM after BC diagnosis may range between several months and many years. [1, 5, 7–9] BC subtype [basal and HER2 receptor status (RS)], the initial Union for International Cancer Control (UICC) stage III–IV of BC and BC adjuvant therapy with Trastuzumab were reported to influence the TI of BC BM [5, 6, 10–12].

As the prognosis after BM occurence is generally poor, the proper and timely management of BM is essential for outcome improvement of affected individuals [8] In this context, the knowledge on the risk factors impacting the timing of metachronous BM might be helpful in timely identification of BM and optimization of the frequency and duration of follow-up care. In particular, as the late occurrence of BM over 10 years after BC diagnosis without BC recurrence and/or extracranial metastases is sporadic, standard BC aftercare does not include routine BM screening after this TI [13, 14]. Previous studies have already shown that the time interval until brain metastasis is relevant for survival after radiosurgery, especially in breast cancer patients [15–17]. Finally, the possible impact of TI on the overall survival (OS) after BM surgery is also controversial, as both significant and nonsignificant associations were previously reported [1, 5, 7, 8, 18].

Therefore, we aimed to identify the predictors associated with TI of BCBM occurrence, as well as to elucidate the impact of TI on OS after BM surgery in a large monocentric series of individuals with metachronous BC BM.

## Material and methods

This study was performed in accordance with the Declaration of Helsinki and approved by the local ethics committee of the University Hospital Essen (local registration number: 17-7855-BO).

### Patient population

All female patients (age ≥ 18 years) who underwent BC BM surgery between January 2008 and December 2019 in a single institution were included. The selection process of individuals for BC BM surgery within the institutional interdisciplinary neuro-oncologic tumor board was reported previously [19, 20]. Patients with synchronous cerebral metastases were excluded from the study.

### Data management

The following patient and tumor characteristics were collected from the electronic health records: patients’ previous medical history (documented comorbidities) and specific laboratory parameters at admission to assess the presence of anemia (hemoglobin), renal function (creatinine), and inflammatory status (white blood cells); BC-related variables: time of BC diagnosis, the type of surgical and (neo-) adjuvant treatment, histopathological features (invasive ductal, invasive lobular), tumor stage, and RS; BM-related variables: time of BM diagnosis, preoperative Karnofsky performance status (KPS) scale, number (singular vs. multiple) and location of BM, RS, and radiographic features in the preoperative magnetic resonance imaging (MRI) as reported previously [20]. In addition, all available follow-up data after BM surgery was recorded to assess the patients’ OS.

A gross total resection was performed in 92 operated metastases. The extent of resection was assessed by postoperative CT imaging and surgical report.

Not all patients underwent adjuvant radiation at our university hospital in Essen, some were treated at other centers, which could not always send a report to us. In summary, 19.4% (n = 18) received whole-brain irradiation, 58.1% (n = 54) received stereotactic irradiation, and in 1.1% (n = 1) it is unclear which radiation was performed. In total, 8 cases (8.6%) were not treated with adjuvant radiation. In 12 cases (12.9%) it is unclear whether and how they were adjuvant radiated.

Of 30 patients with multiple metastases, in 23 of cases (76.7%) one metastasis was removed. In 4 patients (13.3%), two metastases were removed. A maximum of 3 metastases were removed only in 3 cases (3.3%) (see supplementary table E1).

The RS evaluation at our neuropathology department was described elsewhere [19]. In short, immunoreactivity defines estrogen receptor, progesterone receptor, and HER2 positive status. With immunohistochemistry, positive HER2 status is described as HER2 3 + (DAKO score) or HER2 + with HER2 gene amplification detected by fluorescence in situ hybridization. Positive receptor status exists with greater than 1% positive staining of tumor cell nuclei.

### Study endpoints and statistical analysis

The evaluation of the impact of TI on postoperative survival after BM surgery was ***the primary endpoint*** of the present study. First, the outcome-relevant cutoff for TI was identified using the receiver operating characteristic (ROC) curve (see supplementary Figure E1). After the subsequent dichotomization, the impact of TI on OS was analyzed using the Kaplan–Meier survival plots and log-rank test. In addition, univariate Cox regression analysis was performed between all recorded BC and BM characteristics with OS after BM surgery. Finally, the associations with P-value < 0.1 were included in the multivariate Cox regression analysis.

As ***secondary study endpoints***, the associations between the previously defined outcome-relevant TI with BC and BM characteristics were analyzed to identify the predictive factors. In addition, we evaluated the BC and BM parameters associated with the late occurrence of BM, defined as TI ≥ 10 years without other distant metastases or BC recurrence. First, univariate analysis using the Chi-square (χ2 test) or Fisher exact test, as appropriate, was performed. Then, the associations with the P value < 0.1 were included in the multivariable binary logistic regression analysis for the identification of independent associations.

Data were analyzed using SPSS (version 28, SPSS Inc., IBM, Chicago, IL, USA) statistical software. The variables were reported in median values and interquartile ranges (IQR) between 25 and 75%, or as the number of cases (with percentage), as appropriate. The significance level for the p value was set at ≤ 0.05.

## Results

**Fig. 1:**
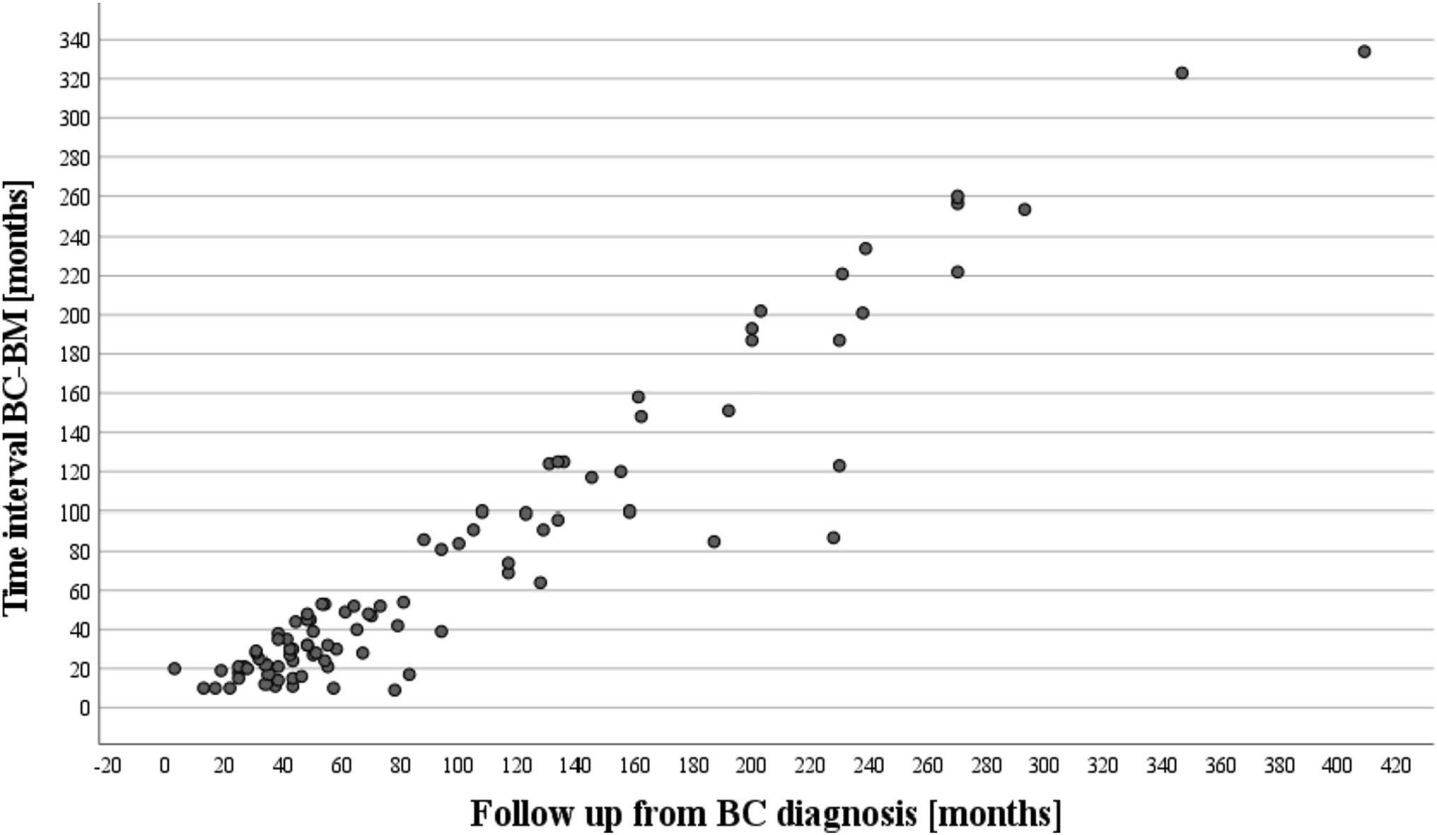
Scatter plot of the TI distribution in the cohort. *BC* breast cancer, *BM* brain metastasis, *TI* time interval

We included 93 female patients with metachronous BM in the final cohort. The median age at the time of BC diagnosis was 52.0 years (IQR 45.5–62.5). The median TI from initial BC diagnosis to BM was 45.0 months (IQR 23.0–100.0) with minimum and maximum TI of 9 and 334 months respectively (Fig. 1). Median OS after BM surgery was 16.0 months (IQR 7.0–33.0). The patients’ characteristics are summarized in Table 1.

**Table 1:** Baseline characteristics of BCBM patients

Parameter	Median (IQR) or No. (%)
Number of patients	93 (100%)
Interval BC to BM (months)	45.0 (23.0–100.0)
BC characteristics	
Age at BC diagnosis (years)	52.0 (45.5–62.5)
Surgical treatment of BC	
Mastectomy / BPS	43 (46.2%)/50 (53.8%)
Systemic treatment of BC	
(Neo-) adjuvant Trastuzumab therapy	25 (26.9%)
Adjuvant BC radiation	62 (66.7%)
Adjuvant Tamoxifen therapy	11 (11.8%)
Histopathology of BC	
Invasive ductal	52 (55.9%)
Invasive lobular	10 (10.8%)
TNM stage	
Initial T stage > *T2*	20 (21.5%)
Initial N stage ≥ *N1*	32 (34.4%)
Initial M stage M1	12 (12.9%)
G stage ≥ G2	49 (52.7%)
UICC stage	
I–II	41 (44.1%)
III–IV	25 (26.9%)
BC subtypes	
Basal (= triple-negative)	19 (20.4%)
LumA (HER2-ER + PR +)	29 (31.2%)
LumB (= triple positive)	10 (10.8%)
HER2 (HER2 + ER-PR-)	21 (22.6%)
Clinical characteristics at BM diagnosis	
Age at BM diagnosis [years]	60.0 (51.5–69.0)
Preoperative seizures	2 (2.2%)
Preoperative KPS score (= 90%)	57 (61.3%)
Preoperative laboratory values	
WBC (≥ *10/nl*)	39 (41.9%)
Hemoglobin (< *12 g/dl*)	15 (16.1%)
Creatinine (> *1.1 mg/dl*)	1 (1.1%)
Pre-existing conditions	
Arterial hypertension	40 (43.0%)
Diabetes mellitus	6 (6.5%)
Hyperuricemia	2 (2.2%)
BM characteristics	
Preoperative MRI	
Tumor necrosis	46 (49.5%)
Edema > 10 mm	67 (72.0%)
Midline shift	13 (14.0%)
Singular/multiple BM	63 (67.7%)/30 (32.3%)
Supratentorial/infratentorial BM	59 (63.4%)/34 (36.6%)
BM receptor status	
HER2 status positive/negative	36 (38.7%)/57 (61.3%)
ER status positive/negative	45 (48.4%)/48 (51.6%)
PR status positive/negative	20 (21.5%)/73 (78.5%)
Identic/converted HER2	70 (75.3%)/9 (9.7%)
Identic/converted ER	58 (62.4%)/21 (22.6%)
Identic/converted PR	54 (58.1%)/25 (26.9%)
Adjuvant brain radiation	73 (90.1%)
Adjuvant systemic therapy after brain radiation	30 (32.3%)

*No* number of cases, *IQR* interquartile ranges 25%–75%, *BC* breast cancer, *BM* brain metastasis, *HER2* human epidermal growth factor receptor 2, *ER* estrogen receptor, *PR* progesterone receptor, *preop* preoperative, *T* tumor size, *N* lymph nodes, *M* distant metastasis, *G* grade of cancer cells, *BPS* breast-preserving surgery, *KPS* Karnofsky Performance Score, *WBC* white blood cells, *UICC* Union for international cancer control, *MRI* magnetic resonance imaging

### Evaluation of the impact of TI on postoperative survival after BM surgery

**Fig. 2:**
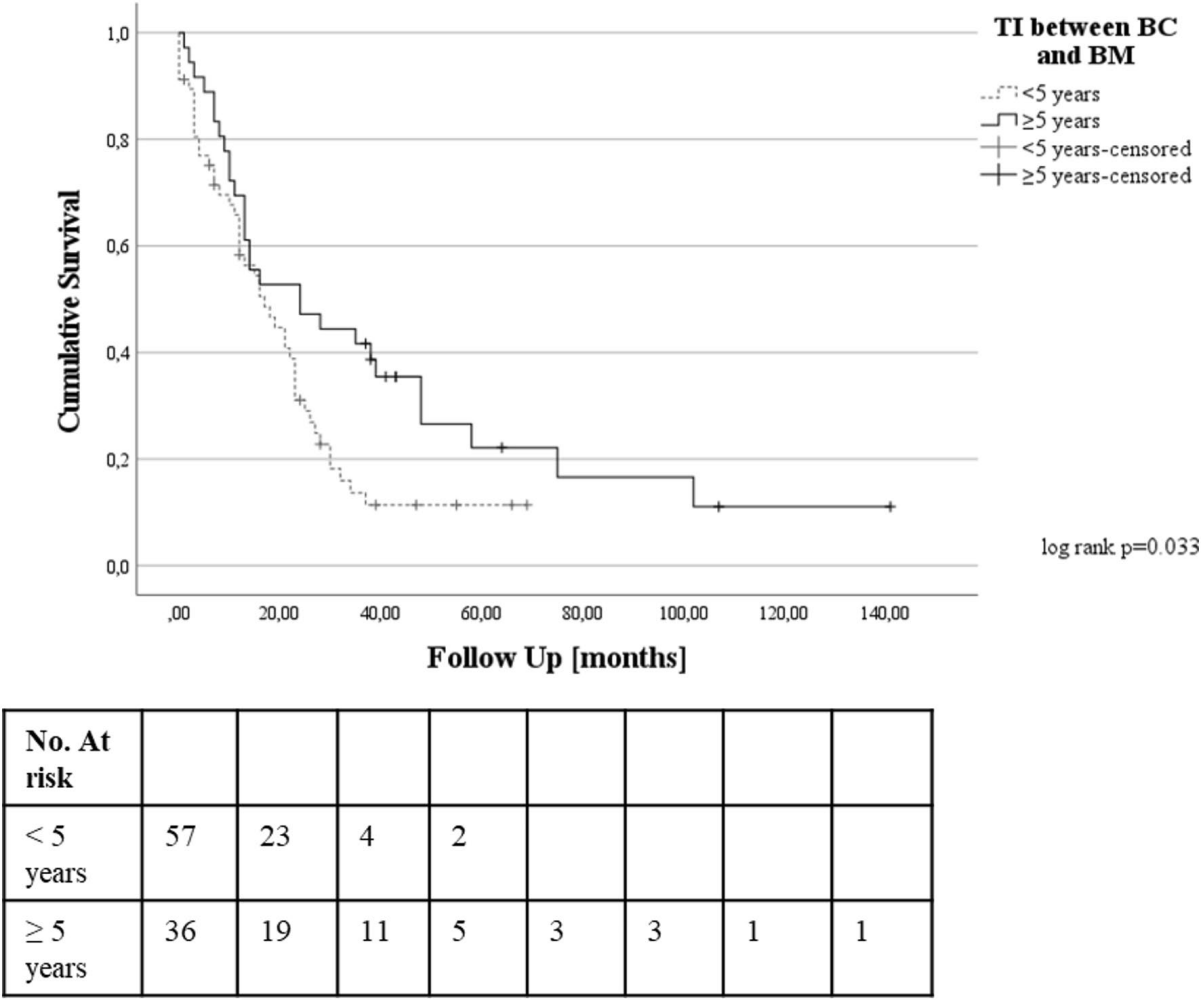
Kaplan Meier curve illustrating the impact of time interval (< 5 years) on OS after BC BM surgery. *BC* breast cancer, *BM* brain metastasis, *TI* time interval, *OS* overall survival., *No* number

Using the ROC curve analysis (Supplementary Fig. E2), TI < 5 years was identified as clinically relevant cutoff for the prediction of OS after BM surgery. Kaplan–Meier survival plot (Fig. 2) showed significant survival differences depending on the above-mentioned TI cutoff.

In the univariate Cox regression analysis, TI < 5 years (p = 0.037) was significantly associated with OS. Moreover, the following parameters were also selected for further evaluation upon the results of univariate analysis (see Supplementary Table E2): age at BC diagnosis ≥ 65 years (p = 0.007), Trastuzumab treatment (p < 0.001), initial N stage N0 (p = 0.027), initial higher G stage (p = 0.077), preoperative KPS < 90% (p = 0.026), preoperative seizure (p = 0.004), tumor necrosis in preoperative MRI (p = 0.037), preoperative leukocytosis (p = 0.073), negative HER2 RS in BM (p = 0.047) and adjuvant brain radiation (p = 0.012).

In the multivariate analysis (see Supplementary Table E3), TI < 5 years [adjusted hazard ratio (aHR) 4.28, 95% confidence interval (CI) 1.46–12.53, p = 0.008], age at BC diagnosis ≥ 65 years (aHR 7.87, 95% CI 1.98–31.33, p = 0.003), initial higher BC G stage (per grade increase: aHR 91.89, 95% CI 3.17–2668.18, p = 0.009), BC treatment with Trastuzumab (aHR 0.06, 95% CI 0.01–0.34, p = 0.001), tumor necrosis in preoperative MRI (aHR 5.15, 95% CI 1.33–19.86, p = 0.017) and adjuvant brain radiation (aHR 0.09, 95% CI 0.01–0.69, p = 0.020) were confirmed as independent predictors of postoperative survival after BM surgery.

### Parameters associated with TI

- *BC characteristics as predictors of outcome-relevant TI (< 5 years)*
  The following BC characteristics were related to shorter TI in univariate analysis (see Supplementary Tables E4): age at BC diagnosis ≥ 65 years (84.2% vs. 55.4%. p = 0.033), invasive ductal BC (71.2% vs. 30.0%, p = 0.026), BC T stage > T2 (80.0% vs. 47.7%, p = 0.028), HER2 RS and basal BC subtype (72.5% vs. 51.3%, p = 0.066, the BC subtypes are shown in supplementary figure E3) and UICC stage III–IV (72.0% vs. 48.8%, p = 0.077).
  The final multivariate analysis, including all above-mentioned parameters showed UICC stage III–IV (adjusted odds ratio [aOR] 4.82, 95% CI 1.10–21.17, p = 0.037) as the only independent predictor of TI < 5 years (see Table 2).
- *Association between TI < 5 years and BM characteristics*
  First, the univariate analysis (see supplementary table E4) detected the following BM features associated with short TI: identic ER status (69.0% vs. 42.9%, p = 0.041), edema ≥ 10 mm (67.2% vs. 35.3%, p = 0.025) and midline shift in the preoperative MRI (92.3% vs. 54.9%, p = 0.013). Of them, midline shift (aOR 10.35, 95% CI 1.09–98.33, p = 0.042) and identic ER status (aOR 4.56, 95%CI 1.35–15.40, p = 0.015) remained independently associated with TI < 5 years in the multivariate analysis (see Table 2).
- *Late occurrence of BM (TI ≥ 10 years)*
  In our cohort, 15(16.1%) patients developed BM 10 years or later after BC diagnosis as first distant metastasis. The univariate analysis (supplementary table E5) identified the association between invasive lobular BC (40.0% vs. 9.6%, p = 0.031) and adjuvant breast radiation (40% vs. 71.8%, p = 0.033) with TI ≥ 10 years (see also Supplementary Table E6 for a detailed comparison of cohort characteristics). In the multivariate analysis, this association remained significant for invasive lobular BC subtype (aOR 9.49, 95% CI 1.47–61.39, p = 0.0018) and adjuvant breast radiation (aOR 0.12, 95% CI 0.02–0.67, p = 0.016, see supplementary table E5).

**Table 2:** Multivariate analysis (binary regression analysis) of predictors of shorter time interval BC-BM (< 5 years)

Parameter	p-value	aOR	95% CI
BC-related characteristics			
Invasive ductal BC subtype	0.311	2.71	0.39–18.59
UICC III–IV	**0.037**	**4.82**	**1.10–21.17**
HER2 and basal BC subtype	0.127	3.00	0.73–12.25
T stage > T2	0.681	1.41	0.28–7.09
Age at BC ≥ 65 years	0.224	3.21	0.49–21.01
BM-related characteristics			
Edema ≥ 10 mm	0.098	2.84	0.83–9.76
Midline shift	**0.042**	**10.35**	**1.09–98.33**
Identic ER status	**0.015**	**4.56**	**1.35–15.40**

Bold indicates significant results

*BC* breast cancer, *BM* brain metastasis, *TI* time interval, *HER2* human epidermal growth factor receptor 2, *ER* estrogen receptor, *T* tumor size, *G* grade of cancer cells, *UICC* Union for international cancer control, *aOR* adjusted odds ratio, *CI* confidence interval, *HER2* subtype: HER2 positive, ER negative, PR negative, basal subtype: triple-negative

**Fig. 3:**
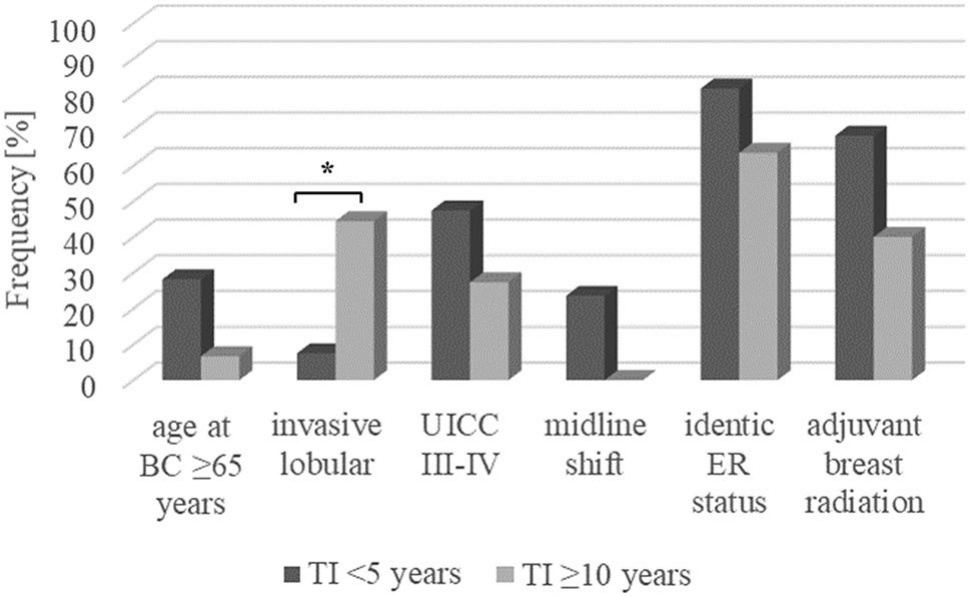
Major differences in BC- and BM-related characteristics between individuals with short (< 5 years) and long (≥ 10 years) TI. *BC* breast cancer, *BM* brain metastasis, *TI* time interval, *ER* estrogen receptor, *UICC* Union for international cancer control, *p < 0.05

Finally, Fig. 3 summarizes the basic differences in the BC/BM characteristics between the individuals with short (< 5 years) and long (≥ 10 years) TI for the occurrence of metachronous BC BM. Additionally, Kaplan Meier curves for the illustration of TI from first BC to BM diagnosis stratified by several parameters are demonstrated in Supplementary Figure E4.

## Discussion

The optimization of diagnostic and treatment strategies on BM improves the prognosis of patients with BC. Metachronous BM might occur at different TI after BC diagnosis, depending on the histological characteristics, initial tumor stadium and BC treatment modalities. Individuals with BM occurring within five years after BC diagnosis show poorer prognosis after BM surgery than their counterparts with later development of BM.

### Impact of TI on OS after BM surgery

There are several acknowledged prognostic markers for postoperative survival after BC BM surgery like the preoperative KPS, BC subtype, age, number of BM and extracranial metastases. [2, 5, 21] As to the possible impact of BM timing on the postoperative course, there are several publications addressing this association, but with conflicting results [1, 5, 7, 8, 18]. Several reasons might underlie this discrepancy such as the differences in baseline characteristics and used TI cutoffs. According to the survival trends in the present cohort, we defined 5 years as the outcome-relevant TI cutoff. Of note, a previous study has already investigated the impact of the 5-year TI on OS in a heterogeneous cohort of BC BM patients, but could not show significant associations [8].

Along with TI, patients’ age and G tumor stage, BC treatment with Trastuzumab, presence of necrosis in BM, and adjuvant brain radiation were independently associated with OS after BM surgery. Except for TI, which remains the contrary discussed survival predictor, other significant results of our study are in line with current evidence in the literature. So, higher age [2, 6] and tumor subtype [2], Trastuzumab therapy [1, 22], brain radiation [18], and tumor necrosis [20] are acknowledged survival predictors for BC BM patients undergoing surgery. Moreover, the relevance of these factors has also been shown for survival prognosis and treatment response of different cancer types, particularly with regard to patients’ age and adjuvant therapy [18]. In addition, the association of tumor necrosis in MRI with poor tumor control after Gamma Knife radiosurgery of lung cancer BM has also been reported [20, 23].

### Shorter TI (< 5 years)—predictors and associations with BC & BM characteristics

For a better understanding of the pathophysiological background of the eventual association between the TI and postoperative survival after BC BM surgery, the evaluation of the link between TI and other patient and tumor characteristics is essential. Accordingly, we analyzed different BC and BM-related characteristics as potential predictors of TI. Consistently with the findings from earlier studies, [5, 6, 8, 10] we could show that the patients’ age, tumor subtype, and stage of initial BC, as well as RS in BM were linked with TI in our univariate analysis. Of BC-related characteristics, only higher tumor stage (UICC III–IV) showed independent associations with shorter TI. The clinical relevance of the initial tumor stage for BM timing [6, 10] and survival prognosis of BC patients in whole [24] was already reported previously. Therefore, the initial BC stage might be the critical factor conditioning the survival effect of TI on the prognosis after BM surgery.

As to the BM-related characteristics, along with radiographic presentation (midline shift on preoperative MRI), an identic ER status in BM was also independently associated with shorter TI in our cohort. The probable link between TI and RS in BC and BM, as well as receptor conversion was also previously reported [9, 25]. BC as heterogenous tumor entity with different tumor cell characteristics and the antihormonal as well as the target therapies could affect complex interactions which result in loss or gain of RS [26]. Several studies described the conversion of RS in distant metastases under adjuvant treatment of BC [9, 27, 28]. In particular, the adjuvant Tamoxifen treatment for ER positive BC might across the blood–brain barrier and influence the ER status in BM [29].

The positive ER status plays an important role in tumor cell differentiation and is related to better prognosis for BC patients [30]. A recent study showed a trend to a shorter disease-free time and poorer prognosis in individuals with identic negative ER status [31]. In addition, an identic ER status was described as poor survival predictor for operated BCBM patients [32]. Our findings confirm the close relationship between the RS in BC and BM with disease progression and prognosis. Further studies are necessary to understand the complex cellular pathways behind the changes in RS, timing of BM and survival prognosis in individuals with BC.

### Late occurrence BM (≥ 10 years): frequency and predictors

Standard tumor aftercare for BC patients does not usually include routine screening for BM. Brain MRI is indicated in individuals with neurological symptoms, initial tumor stage IV, or triple-negative BC [33–35]. In addition, BM in BC patients as a first distant metastasis after more than 10 years is very rare [13, 14, 36]. Generally, BC patients with a stable disease without distant metastases and/or BC recurrence after 10 years are not closely followed up, especially with regard to distant metastases [37, 38].

In this context, the analysis of the rate and risk factors of late occurrence of BC BM (≥ 10 years) is of particular clinical interest. In our cohort, late BM were common (16.1%) in BC patients. We identified the invasive lobular BC subtype and adjuvant breast radiation as significant predictors of TI ≥ 10 years. Interestingly, invasive ductal subtype of BC showed associations with the risk of early BM occurrence (< 5 years) in our univariate analysis. The possible link between histopathological features of BC and further disease course has already been discussed previously. So, longer disease-free survival after the diagnosis of invasive lobular BC was reported already in a publication from 1994 [39]. However, recent studies could not identify survival differences between invasive lobular and ductal BC subtypes [40, 41]. The observed differences in the timing of BM might be related to the biological features of the BC subtypes. In particular, the invasive ductal BC might be prone to an earlier occurrence of distant metastasis due to their increased collective epithelial invasion, which is triggered by E-cadherin expression [42, 43]. In turn, the invasive lobular BC is characterized by higher rate of cell individualization resulting in slower development of distant metastasis [42, 43]. Our findings support the impact of BC subtype on the timing of BM.

Finally, there was an association between adjuvant breast radiation and late BM occurrence in the present study. The background of this link might be related to the effect of initial surgical strategy of BC treatment. So, individuals with late BM showed a higher proportion of mastectomy, after which radiation was not given. In case of breast-conserving surgery, residual tumor cells might sprout into the vascular and lymph nodal system and influence the risk and timing of distant metastases [44, 45]. This aspect might have led in the case of the late metastasized patients, most of whom were treated with mastectomy, to the presence of fewer residual tumor cells and thus the occurrence of distant metastases and thus brain metastases at a later time. The adjuvant radiation could promote the molecular changes in the residual tumor burden with the possibility of development of aggressiveness and resistance of BC [46, 47]. This circumstance might explain that in our group of late BM occurrence, the lack of breast irradiation resulted in fewer molecular changes and contributed to less early aggressiveness of BC. The irradiation of tumor cells increases the growth capacity and the transformation process. In this way, the tumorigenic effect is supported by the radiation [48]. It has been also reported that irradiation leads to greater heterogeneity of cells in breast tissue [49]. Although the exact role of residual tumor cells in the aggressiveness of BC BM is unknown [50], the described pathophysiological processes after initial BC treatment might condition the effect of adjuvant breast radiation on the timing of BM.

In summary, the reported predictors of TI between BC and BM underline the importance of an adjusted follow-up strategy, not limited to 10 years, depending on initial tumor and patient characteristics and previous BC treatment.

## Limitations

The major limitations of this study were the retrospective and monocentric design as well as the incompleteness of patient and follow-up data. Moreover, our analyses were related to the specific group of BC patients with BM who received surgical therapy for BM that limits the generalizability of our study results to non-surgically treated BC BM patients. Finally, the relatively small sample size has limited the statistical power of the study results.

## Conclusion

TI between BC and BM diagnosis impacts the survival prognosis after BM surgery. Individuals with BM occurring within 5 years after BC diagnosis are more likely to present with midline shift in preoperative MRI, identic ER status in BM, and show poorer OS. The initial BC tumor stage UICC III–IV was related to the risk of shorter TI (< 5 years). Moreover, later occurrence of BM (≥ 10 years) without any other distant metastases was common (16.1%) in our surgical cohort. This circumstance underlines the need for an individualized follow-up strategy of BC patients, not limited to 10 years. Especially, the patients with invasive lobular BC type and without initial adjuvant breast radiation were prone to later onset of first distant metastases in brain. Further comparative studies with larger data samples and inclusion of different BC subpopulations are needed for the confirmation of the presented study results.

## Supplementary Information

Below is the link to the electronic supplementary material.

Supplementary file1 (DOCX 182 kb)

## Data Availability

The data presented in the study are available on request from the corresponding author. All data are not publicly available due to ethical restrictions.

## Abbreviations

aOR: Adjusted odds ratios
aHR: Adjusted hazard ratios
AUC: Areas under the curve
BC: Breast cancer
BM: Brain metastases
CI: Confidence interval
ER: Estrogen receptor
G: Grade of cancer cells
HER2: Human epidermal growth factor receptor 2
IQR: Interquartile ranges
KPS: Karnofsky Performance Status
MRI: Magnetic resonance imaging
OS: Overall survival
PR: Progesterone receptor
ROC: Receiver operating characteristic
RS: Receptor status
T N M stage: T: Tumor size, N: Lymph nodes, M: distant metastasis
TI: Time interval
UICC: Union for international cancer control

## References

[1] Rostami R, Mittal S, Rostami P, Tavassoli F, Jabbari B (2016). Brain metastasis in breast cancer: a comprehensive literature review. J Neurooncol.

[2] Sperduto PW, Kased N, Roberge D, Xu Z, Shanley R, Luo X, Sneed PK, Chao ST, Weil RJ, Suh J, Bhatt A, Jensen AW, Brown PD, Shih HA, Kirkpatrick J, Gaspar LE, Fiveash JB, Chiang V, Knisely JP, Sperduto CM, Lin N, Mehta M (2012). Summary report on the graded prognostic assessment: an accurate and facile diagnosis-specific tool to estimate survival for patients with brain metastases. J Clin Oncol.

[3] Li X, Huang R, Ma L, Liu S, Zong X (2019). Locoregional surgical treatment improves the prognosis in primary metastatic breast cancer patients with a single distant metastasis except for brain metastasis. Breast.

[4] Press DJ, Miller ME, Liederbach E, Yao K, Huo D (2017). De novo metastasis in breast cancer: occurrence and overall survival stratified by molecular subtype. Clin Exp Metastasis.

[5] Sperduto PW, Kased N, Roberge D, Chao ST, Shanley R, Luo X, Sneed PK, Suh J, Weil RJ, Jensen AW, Brown PD, Shih HA, Kirkpatrick J, Gaspar LE, Fiveash JB, Chiang V, Knisely JP, Sperduto CM, Lin N, Mehta M (2013). The effect of tumor subtype on the time from primary diagnosis to development of brain metastases and survival in patients with breast cancer. J Neurooncol.

[6] Leone JP, Lee AV, Brufsky AM (2015). Prognostic factors and survival of patients with brain metastasis from breast cancer who underwent craniotomy. Cancer Med.

[7] Ahn HK, Park YH, Lee SJ, Park S, Maeng CH, Park W, Choi DH, Hur SJ, Ahn JS, Im YH (2013). Clinical implication of time to brain metastasis (TTBM) according to breast cancer subtypes. Springerplus.

[8] Lee SS, Ahn JH, Kim MK, Sym SJ, Gong G, Ahn SD, Kim SB, Kim WK (2008). Brain metastases in breast cancer: prognostic factors and management. Breast Cancer Res Treat.

[9] Timmer M, Werner JM, Rohn G, Ortmann M, Blau T, Cramer C, Stavrinou P, Krischek B, Mallman P, Goldbrunner R (2017). Discordance and conversion rates of progesterone-, estrogen-, and HER2/neu-receptor status in primary breast cancer and brain metastasis mainly triggered by hormone therapy. Anticancer Res.

[10] Saraf A, Grubb CS, Hwang ME, Tai CH, Wu CC, Jani A, Lapa ME, Andrews JIS, Vanderkelen S, Isaacson SR, Sonabend AM, Sheth SA, McKhann GM, Sisti MB, Bruce JN, Cheng SK, Connolly EP, Wang TJC (2017). Breast cancer subtype and stage are prognostic of time from breast cancer diagnosis to brain metastasis development. J Neurooncol.

[11] Dawood S, Broglio K, Esteva FJ, Ibrahim NK, Kau SW, Islam R, Aldape KD, Yu TK, Hortobagyi GN, Gonzalez-Angulo AM (2008). Defining prognosis for women with breast cancer and CNS metastases by HER2 status. Ann Oncol.

[12] Bendell JC, Domchek SM, Burstein HJ, Harris L, Younger J, Kuter I, Bunnell C, Rue M, Gelman R, Winer E (2003). Central nervous system metastases in women who receive trastuzumab-based therapy for metastatic breast carcinoma. Cancer.

[13] Ogawa K, Yoshii Y, Nishimaki T, Tamaki N, Miyaguni T, Tsuchida Y, Kamada Y, Toita T, Kakinohana Y, Tamaki W, Iraha S, Adachi G, Hyodo A, Murayama S (2008). Treatment and prognosis of brain metastases from breast cancer. J Neurooncol.

[14] Yamada SM, Tomita Y, Shibui S, Kurokawa T, Baba Y (2017). A Case of breast cancer brain metastasis with a 16-year time interval without evidence of cancer recurrence. J Breast Cancer.

[15] Soike MH, McTyre ER, Hughes RT, Farris M, Cramer CK, LeCompte MC, Lanier CM, Ruiz J, Su J, Watabe K, Bourland JD, Munley MT, O'Neill S, Laxton AW, Tatter SB, Chan MD (2018). Initial brain metastasis velocity: does the rate at which cancers first seed the brain affect outcomes?. J Neurooncol.

[16] Yamamoto M, Aiyama H, Koiso T, Watanabe S, Kawabe T, Sato Y, Higuchi Y, Kasuya H, Barfod BE (2019). Applicability and limitations of a recently-proposed prognostic grading metric, initial brain metastasis velocity, for brain metastasis patients undergoing stereotactic radiosurgery. J Neurooncol.

[17] Yamamoto M, Serizawa T, Nagano O, Aoyagi K, Higuchi Y, Sato Y, Kasuya H, Barfod BE (2020). Three-institution study on applicability of initial brain metastasis velocity for breast cancer brain metastasis patients undergoing stereotactic radiosurgery. J Neurooncol.

[18] Kim HJ, Im SA, Keam B, Kim YJ, Han SW, Kim TM, Oh DY, Kim JH, Lee SH, Chie EK, Han W, Kim DW, Kim TY, Noh DY, Heo DS, Park IA, Bang YJ, Ha SW (2012). Clinical outcome of central nervous system metastases from breast cancer: differences in survival depending on systemic treatment. J Neurooncol.

[19] Michel A, Oppong MD, Rauschenbach L, Pierscianek D, Dinger TF, Schmidt T, Hense J, Pottgen C, Kimmig R, Ahmadipour Y, Ozkan N, Muller O, Junker A, Sure U, Jabbarli R, El Hindy N (2021). HER2 Receptor conversion is a strong survival predictor in patients with breast cancer brain metastases. World Neurosurg.

[20] Michel A, Dinger T, Darkwah Oppong M, Rauschenbach L, Deuschl C, Ahmadipour Y, Pierscianek D, Wrede K, Hense J, Pottgen C, Iannaccone A, Kimmig R, Sure U, Jabbarli R (2022). Radiographic markers of breast cancer brain metastases: relation to clinical characteristics and postoperative outcome. Acta Neurochir (Wien).

[21] Sperduto PW, Mesko S, Li J, Cagney D, Aizer A, Lin NU, Nesbit E, Kruser TJ, Chan J, Braunstein S, Lee J, Kirkpatrick JP, Breen W, Brown PD, Shi D, Shih HA, Soliman H, Sahgal A, Shanley R, Sperduto W, Lou E, Everett A, Boggs DH, Masucci L, Roberge D, Remick J, Plichta K, Buatti JM, Jain S, Gaspar LE, Wu CC, Wang TJC, Bryant J, Chuong M, Yu J, Chiang V, Nakano T, Aoyama H, Mehta MP (2020). Beyond an Updated Graded Prognostic Assessment (Breast GPA): a prognostic index and trends in treatment and survival in breast cancer brain metastases from 1985 to today. Int J Radiat Oncol Biol Phys.

[22] Bartsch R, Rottenfusser A, Wenzel C, Dieckmann K, Pluschnig U, Altorjai G, Rudas M, Mader RM, Poetter R, Zielinski CC, Steger GG (2007). Trastuzumab prolongs overall survival in patients with brain metastases from Her2 positive breast cancer. J Neurooncol.

[23] Huang CY, Lee CC, Yang HC, Lin CJ, Wu HM, Chung WY, Shiau CY, Guo WY, Pan DH, Peng SJ (2020). Radiomics as prognostic factor in brain metastases treated with Gamma Knife radiosurgery. J Neurooncol.

[24] Wang R, Zhu Y, Liu X, Liao X, He J, Niu L (2019). The Clinicopathological features and survival outcomes of patients with different metastatic sites in stage IV breast cancer. BMC Cancer.

[25] Bachmann C, Grischke EM, Fehm T, Staebler A, Schittenhelm J, Wallwiener D (2013). CNS metastases of breast cancer show discordant immunohistochemical phenotype compared to primary. J Cancer Res Clin Oncol.

[26] McDermott SP, Wicha MS (2010). Targeting breast cancer stem cells. Mol Oncol.

[27] Hulsbergen AFC, Claes A, Kavouridis VK, Ansaripour A, Nogarede C, Hughes ME, Smith TR, Brastianos PK, Verhoeff JJC, Lin NU, Broekman MLD (2020). Subtype switching in breast cancer brain metastases: a multicenter analysis. Neuro Oncol.

[28] Sperduto PW, Mesko S, Li J, Cagney D, Aizer A, Lin NU, Nesbit E, Kruser TJ, Chan J, Braunstein S, Lee J, Kirkpatrick JP, Breen W, Brown PD, Shi D, Shih HA, Soliman H, Sahgal A, Shanley R, Sperduto W, Lou E, Everett A, Boggs DH, Masucci L, Roberge D, Remick J, Plichta K, Buatti JM, Jain S, Gaspar LE, Wu CC, Wang TJC, Bryant J, Chuong M, Yu J, Chiang V, Nakano T, Aoyama H, Mehta MP (2020). Estrogen/progesterone receptor and HER2 discordance between primary tumor and brain metastases in breast cancer and its effect on treatment and survival. Neuro Oncol.

[29] Lien EA, Wester K, Lonning PE, Solheim E, Ueland PM (1991). Distribution of tamoxifen and metabolites into brain tissue and brain metastases in breast cancer patients. Br J Cancer.

[30] Braun L, Mietzsch F, Seibold P, Schneeweiss A, Schirmacher P, Chang-Claude J, Peter Sinn H, Aulmann S (2013). Intrinsic breast cancer subtypes defined by estrogen receptor signalling-prognostic relevance of progesterone receptor loss. Mod Pathol.

[31] Zhu YY, Si W, Ji TF, Guo XQ, Hu Y, Yang JL (2016). The variation and clinical significance of hormone receptors and Her-2 status from primary to metastatic lesions in breast cancer patients. Tumour Biol.

[32] Basak Oven Ustaalioglu B, Aker Vardar F, Bilici A, Gurleyik G, Erkol B, Kefeli U, Aliustaoglu M (2014). Clinical importance of discordance of hormone receptors and Her2/neu status after neoadjuvant chemotherapy in breast cancer. J BUON.

[33] Le Rhun E, Guckenberger M, Smits M, Dummer R, Bachelot T, Sahm F, Galldiks N, de Azambuja E, Berghoff AS, Metellus P, Peters S, Hong YK, Winkler F, Schadendorf D, van den Bent M, Seoane J, Stahel R, Minniti G, Wesseling P, Weller M, Preusser M, EANO Executive Board and ESMO Guidelines Committee (2021). EANO-ESMO Clinical Practice Guidelines for diagnosis, treatment and follow-up of patients with brain metastasis from solid tumours. Ann Oncol.

[34] Niikura N, Hayashi N, Masuda N, Takashima S, Nakamura R, Watanabe K, Kanbayashi C, Ishida M, Hozumi Y, Tsuneizumi M, Kondo N, Naito Y, Honda Y, Matsui A, Fujisawa T, Oshitanai R, Yasojima H, Tokuda Y, Saji S, Iwata H (2014). Treatment outcomes and prognostic factors for patients with brain metastases from breast cancer of each subtype: a multicenter retrospective analysis. Breast Cancer Res Treat.

[35] Ramakrishna N, Temin S, Chandarlapaty S, Crews JR, Davidson NE, Esteva FJ, Giordano SH, Kirshner JJ, Krop IE, Levinson J, Modi S, Patt DA, Perlmutter J, Winer EP, Lin NU (2018). Recommendations on disease management for patients with advanced human epidermal growth factor receptor 2-positive breast cancer and brain Metastases: ASCO clinical practice guideline update. J Clin Oncol.

[36] Pestalozzi BC, Zahrieh D, Price KN, Holmberg SB, Lindtner J, Collins J, Crivellari D, Fey MF, Murray E, Pagani O, Simoncini E, Castiglione-Gertsch M, Gelber RD, Coates AS, Goldhirsch, International Breast Cancer Study G (2006). Identifying breast cancer patients at risk for central nervous system (CNS) metastases in trials of the international breast cancer study group (IBCSG). Ann Oncol.

[37] Saphner T, Tormey DC, Gray R (1996). Annual hazard rates of recurrence for breast cancer after primary therapy. J Clin Oncol.

[38] Leitliniendetailansicht AdWMFeV (2021) Interdisziplinäre S3-Leitlinie für die Früherkennung, Diagnostik, Therapie und Nachsorge des Mammakarzinoms. AWMF online Das Portal der wissenschaftlichen Medien Langversion 4.4 – Juni 2021, AWMF-Registernummer: 032-045OL

[39] Silverstein MJ, Lewinsky BS, Waisman JR, Gierson ED, Colburn WJ, Senofsky GM, Gamagami P (1994). Infiltrating lobular carcinoma Is it different from infiltrating duct carcinoma?. Cancer.

[40] Viale G, Rotmensz N, Maisonneuve P, Orvieto E, Maiorano E, Galimberti V, Luini A, Colleoni M, Goldhirsch A, Coates AS (2009). Lack of prognostic significance of “classic” lobular breast carcinoma: a matched, single institution series. Breast Cancer Res Treat.

[41] Flores-Diaz D, Arce C, Flores-Luna L, Reynoso-Noveron N, Lara-Medina F, Matus JA, Bargallo-Rocha E, Perez V, Villarreal-Garza C, Cabrera-Galeana P, Mohar A (2019). Impact of invasive lobular carcinoma on long-term outcomes in Mexican breast cancer patients. Breast Cancer Res Treat.

[42] Khalil AA, Ilina O, Gritsenko PG, Bult P, Span PN, Friedl P (2017). Collective invasion in ductal and lobular breast cancer associates with distant metastasis. Clin Exp Metastasis.

[43] Friedl P, Locker J, Sahai E, Segall JE (2012). Classifying collective cancer cell invasion. Nat Cell Biol.

[44] Tachtsidis A, McInnes LM, Jacobsen N, Thompson EW, Saunders CM (2016). Minimal residual disease in breast cancer: an overview of circulating and disseminated tumour cells. Clin Exp Metastasis.

[45] Dupont VN, Gentien D, Oberkampf M, De Rycke Y, Blin N (2007). A gene expression signature associated with metastatic cells in effusions of breast carcinoma patients. Int J Cancer.

[46] Olson P, Hanahan D (2009). Cancer. Breaching the cancer fortress. Science.

[47] Stewart DJ, Chiritescu G, Dahrouge S, Banerjee S, Tomiak EM (2007). Chemotherapy dose–response relationships in non-small cell lung cancer and implied resistance mechanisms. Cancer Treat Rev.

[48] Repulles J, Anglada T, Soler D, Ramirez JC, Genesca A, Terradas M (2019). Radiation-induced malignant transformation of preneoplastic and normal breast primary epithelial cells. Mol Cancer Res.

[49] Li S, Zhou J, Wu H, Lu Q, Tai Y, Liu Q, Wang C (2018). Oncogenic transformation of normal breast epithelial cells co-cultured with cancer cells. Cell Cycle.

[50] Narod SA, Sopik V (2018). Is invasion a necessary step for metastases in breast cancer?. Breast Cancer Res Treat.

