# Supplementary material for: Time interval between the diagnosis of breast cancer and brain metastases impacts prognosis after metastasis surgery
Source: J Neurooncol. 2022 Jun 7;159(1):53–63. doi: 10.1007/s11060-022-04043-2 (PMC9325855; doi:10.1007/s11060-022-04043-2)
Supplement: Supplementary file 1 — Supplementary file1 (DOCX 182 kb) [file 11060_2022_4043_MOESM1_ESM.docx]

**Journal of Neuro-Oncology**

**Time interval between the diagnosis of breast cancer and brain metastases impacts prognosis after metastasis surgery**

*Anna Michel ^1,5,6^,* *Thiemo Florin Dinger ^1,5,6^, Alejandro N. Santos^1,5,6^, Daniela Pierscianek ^1,5,6^, Marvin Darkwah Oppong ^1,5,6^, Yahya Ahmadipour ^1,5,6^, Philipp Dammann ^1,5,6^, Karsten H. Wrede ^1,5,6^, Jörg Hense ^2,6^, Christoph Pöttgen ^3,6^, Antonella Iannaccone ^4,6^, Rainer Kimmig ^4,6^, Ulrich Sure ^1,5,6^, Ramazan Jabbarli ^1,5,6^*

**Affiliation:**

1. Department of Neurosurgery and Spine Surgery, University Hospital Essen, Essen, Germany
2. Department of Medical Oncology, University Hospital Essen, Essen, Germany
3. Department of Radiotherapy, University Hospital Essen, Essen, Germany
4. Department of Obstetrics and Gynecology, University Hospital Essen, Essen, Germany
5. Center for Translational Neuro- & Behavioral Sciences (C-TNBS), University Duisburg Essen, Germany
6. German Cancer Consortium (DKTK) Partner Site, University Hospital Essen, 45147 Essen, Germany.

**Corresponding author:**

Dr. med. Anna Michel

Department of Neurosurgery and Spine surgery, University Hospital Essen, University Duisburg-Essen, Hufelandstraße 55, 45147 Essen, Germany

E-Mail: anna.michel@uk-essen.de

Telephone Number: 00492017231230/ Fax Number: 00492017231220

**ONLINE SUPPLEMENTS**

**Supplementary Table E1:** Multiple metastases and their extent of resection

| **No. of BM** | **No. of patients** | **No. of resected BM (No. of patients)** |
| --- | --- | --- |
| 2 | 17 | 1 (14), 2(3) |
| 3 | 10 | 1 (8), 2 (1), 3 (1) |
| 4 | 1 | 1 (1) |
| 5 | 0 | 0 |
| 6 | 0 | 0 |
| 7 | 1 | 3 (1) |
| 8 | 1 | 3 (1) |

Abbreviations: BM: brain metastasis, No.: number

**Supplementary Table E2:** Univariate Cox regression analysis of predictors related to OS after BM surgery

| **Parameter** | **p-value** | **HR** | **95% CI** |
| --- | --- | --- | --- |
| TI <5 years | **0.037** | **1.69** | 1.03-2.76 |
| Age at BC diagnosis ≥65 years | **0.007** | **2.15** | 1.23-3.74 |
| Age at BM ≥65 years | **0.041** | **1.62** | 1.02-2.57 |
| Invasive ductal BC subtype | 0.517 | 1.31 | 0.58-2.94 |
| HER2 and basal BC subtype | 0.187 | 0.71 | 0.43-1.18 |
| Mastectomy | 0.922 | 1.02 | 0.65-1.62 |
| (Neo-) adjuvant Trastuzumab treatment | **<0.001** | **0.37** | 0.21-0.66 |
| Adjuvant BC radiation | 0.338 | 1.27 | 0.78-2.08 |
| Adjuvant Tamoxifen | 0.664 | 0.86 | 0.43-1.73 |
| T stage >T2 | 0.387 | 1.31 | 0.71-2.44 |
| N stage N0 | **0.027** | **1.94** | 1.10-3.49 |
| M1 stage | 0.928 | 0.97 | 0.46-2.03 |
| UICC III-IV | 0.818 | 1.07 | 0.61-1.88 |
| Higher G stage | **0.077** |  |  |
|  | **0.025** | **5.73** | 1.25-26.35 |
|  | **0.951** | **1.02** | 0.53-1.97 |
| Number of BM >1 | 0.186 | 1.38 | 0.86-2.24 |
| Infratentorial BM | 0.355 | 1.25 | 0.78-.1.99 |
| Preop. KPS <90 | **0.026** | **1.69** | 1.07-2.68 |
| Preop. seizure | **0.004** | **8.93** | 2.03-39.29 |
| Preop. MRI tumor necrosis | **0.037** | **1.72** | 1.03-2.87 |
| Preop. MRI edema ≥10mm | 0.544 | 1.21 | 0.65-2.24 |
| Preop. MRI midline shift | 0.420 | 1.30 | 0.69-2.45 |
| Preop. Leukocytosis (≥10/nl) | **0.073** | **1.56** | 0.96-2.54 |
| Preop. Anemia (hemoglobin <12g/dl) | 0.559 | 1.22 | 0.62-2.41 |
| Preop. Creatinine ≥1.1mg/dl | 0.116 | 5.06 | 0.67-38.32 |
| Identic HER2 RS | 0.472 | 0.75 | 0.34-1.65 |
| Identic ER status | 0.253 | 1.42 | 0.78-2.60 |
| Identic PR status | 0.924 | 0.97 | 0.56-1.69 |
| Negative HER2 RS in BM | **0.047** | **1.63** | 1.01-2.63 |
| Negative ER status in BM | 0.477 | 1.18 | 0.75-1.87 |
| Negative PR status in BM | 0.252 | 0.74 | 0.44-1.24 |
| Late occurrence BM patients (≥10 years) | 0.760 | 0.91 | 0.49-1.69 |
| Adjuvant brain radiation | **0.012** | **0.36** | 0.16-0.80 |
| Adjuvant systemic therapy and brain radiation (*) | **0.055** | **0.59** | 0.34-1.01 |
| Extracranial metastases | 0.345 | 1.25 | 0.79-2.00 |

Abbreviations: BC: breast cancer, BM: brain metastasis, HER2: human epidermal growth factor receptor 2, ER: estrogen receptor, PR: progesterone receptor, Ki67: marker of proliferation, T: tumor size, N: lymph nodes, M: distant metastasis, G: grade of cancer cells, HR: hazard ratio, CI: confidence interval, RS: receptor status, preop.: preoperative, CE: contrast enhancement, KPS: Karnofsky Performance Score, OR: odds ratio, CI: confidence interval, *: excluded for multivariate analysis.

**Supplementary Table E3:** Multivariate Cox regression analysis of predictors of OS after BM surgery

| **Parameter** | **p-value** | **aHR** | **95% CI** |
| --- | --- | --- | --- |
| TI <5 years | **0.008** | **4.28** | **1.46-12.53** |
| Age at BC diagnosis ≥65 years | **0.003** | **7.87** | **1.98-31.33** |
| Higher G stage | **0.015** |  |  |
| G1 vs. G2 and G3 | **0.009** | **91.89** | **3.17-2668.18** |
| G2 vs. G1 and G3 | **0.026** | **3.61** | **1.16-11.18** |
| (Neo-) adjuvant Trastuzumab treatment | **0.001** | **0.06** | **0.01-0.34** |
| N stage N0 | 0.844 | 1.10 | 0.41-2.94 |
| Preoperative KPS score <90% | 0.246 | 1.88 | 0.65-5.44 |
| Preoperative WBC≥10/nl | 0.450 | 1.62 | 0.47-5.61 |
| Tumor necrosis in MRI | **0.017** | **5.15** | **1.33-19.86** |
| Negative HER2 status in BM | 0.147 | 2.78 | 0.70-11.05 |
| Adjuvant brain radiation | **0.020** | **0.09** | **0.01-0.69** |

Abbreviations: BC: breast cancer, BM: brain metastasis, HER2: human epidermal growth factor receptor 2, N: lymph nodes, G: grade of cancer cells, KPS: Karnofsky Performance score, MRI: Magnetic resonance imaging, OS: overall survival, aHR: adjusted hazard ratio, CI: confidence interval; WBC: white blood cells.

**Supplementary Table E4:** Univariate analysis (chi-square test) of predictors of short TI (<5 years)

| **Parameter** | **p-value** | **OR** | **95% CI** |
| --- | --- | --- | --- |
| **BC-related characteristics** | | | |
| Age at BC diagnosis ≥65 years | **0.033** | 4.29 | 1.15-16.00 |
| Invasive ductal BC subtype | **0.026** | **5.76** | 1.31-25.27 |
| HER2 and basal BC subtype | **0.066** | **2.51** | 0.98-6.39 |
| Breast-preserving surgery | 1.000 | 1.07 | 0.46-2.46 |
| (Neo-) adjuvant Trastuzumab treatment | 0.814 | 1.17 | 0.45-3.03 |
| Not adjuvant radiation | 0.659 | 1.23 | 0.51-2.95 |
| Not adjuvant Tamoxifen | 0.100 | 3.20 | 0.86-11.84 |
| T stage >T2 | **0.028** | **4.38** | 1.26-15.22 |
| N stage N0 | 1.000 | 1.04 | 0.37-2.89 |
| M1 stage | 0.759 | 1.33 | 0.37-4.80 |
| UICC III-IV | **0.077** | **2.70** | 0.93-7.84 |
| G stage ≥G2 | 0.149 | 1.11 | 0.96-1.29 |
| **BM-related characteristics** | | | |
| Age at BM ≥65 years | 0.518 | 1.37 | 0.59-3.21 |
| Number of BM >1 | 0.823 | 1.14 | 0.46-2.79 |
| Supratentorial BM | 0.826 | 1.18 | 0.50-2.79 |
| Preop. KPS <90 | 0.513 | 1.46 | 0.61-3.47 |
| Not preop. Seizure | 1.000 | 1.60 | 0.10-26.41 |
| Preop. MRI tumor necrosis | 0.660 | 1.24 | 0.52-2.99 |
| Preop. MRI edema ≥10mm | **0.025** | **3.75** | 1.23-11.47 |
| Preop. MRI midline shift | **0.013** | **9.85** | 1.21-79.83 |
| Preop. Leukocytosis (≥10/nl) | 0.186 | 1.89 | 0.77-4.62 |
| Preop. Hemoglobin <12g/dl | 0.248 | 0.49 | 0.16-1.50 |
| Preop. Creatinine >1.1mg/dl | 0.393 | **-** | - |
| HER2 identic | 0.289 | 2.25 | 0.55-9.15 |
| ER identic | **0.041** | **2.96** | 1.06-8.28 |
| PR identic | 0.225 | 1.85 | 0.70-4.86 |
| Positive HER2 RS in BM | 0.275 | 1.78 | 0.74-4.29 |
| Negative ER status in BM | 0.201 | 1.87 | 0.80-4.38 |
| Negative PR status in BM | 0.607 | 1.40 | 0.51-3.79 |

Abbreviations: TI: time interval; BC: breast cancer, BM: brain metastasis, HER2: human epidermal growth factor receptor 2, ER: estrogen receptor, PR: progesterone receptor, Ki67: marker of proliferation, T: tumor size, N: lymph nodes, M: distant metastasis, G: grade of cancer cells, RS: receptor status, RC: receptor conversion, preop.: preoperative, CE: contrast enhancement, KPS: Karnofsky Performance Score, OR: odds ratio, CI: confidence interval.

**Supplementary Table E5:** Univariate (chi-square test) and multivariate analysis of BC- and BM-related predictors for patients with late occurrence BM (≥10 years)

| **Parameter** | **p-value** | **OR** | **95% CI** |
| --- | --- | --- | --- |
| **BC-related characteristics (univariate analysis)** | | | |
| Age at BC diagnosis <65 years | 0.291 | 4.20 | 0.52-34.16 |
| Invasive lobular BC subtype | **0.031** | **6.27** | 1.31-29.99 |
| HER2 and basal BC subtype | 0.115 | 3.18 | 0.78-13.04 |
| BC Ablatio as surgical treatment | 1.000 | 1.02 | 0.34-3.09 |
| (Neo-) adjuvant Trastuzumab treatment | 0.752 | 0.64 | 0.16-2.47 |
| Adjuvant radiation | **0.033** | **0.26** | 0.08-0.82 |
| Adjuvant Tamoxifen | 0.376 | 0.46 | 0.11-1.97 |
| T stage ≤T2 | 0.152 | 4.89 | 0.58-41.53 |
| N stage ≥N1 | 0.737 | 1.39 | 0.35-5.51 |
| M1 stage | 1.000 | 1.03 | 0.20-5.26 |
| UICC I-II | 0.513 | 1.78 | 0.42-7.45 |
| G stage | 0.127 | - | - |
| **BM-related characteristics (univariate analysis)** | | | |
| Age at BM ≥65 years | 0.576 | 1.40 | 0.46-4.26 |
| Number of BM >1 | 0.551 | 1.50 | 0.48-4.69 |
| Infratentorial BM | 0.776 | 1.19 | 0.38-3.69 |
| Preoperative KPS ≥90% | 0.776 | 1.32 | 0.41-4.23 |
| Preoperative seizures | 1.000 | - | - |
| Tumor necrosis in MRI | 0.773 | 0.80 | 0.25-2.51 |
| Edema <10mm in MRI | 0.468 | 1.75 | 0.48-6.48 |
| Midline shift in MRI | 0.113 | **-** | - |
| Preoperative WBC ≥10/nl | 0.777 | 0.75 | 0.24-2.33 |
| Preoperative Hemoglobin <12g/dl | 0.454 | 1.95 | 0.53-7.25 |
| Preoperative Creatinine >1.1mg/dl | 1.000 | - | - |
| HER2 converted | 0.106 | 3.88 | 0.81-18.61 |
| ER converted | 0.470 | 1.71 | 0.45-6.58 |
| PR converted | 0.311 | 2.00 | 0.55-7.31 |
| Negative HER2 RS in BM | 0.391 | 1.91 | 0.56-6.55 |
| Positive ER status in BM | 0.264 | 2.11 | 0.66-6.73 |
| Positive PR status in BM | 0.301 | 2.10 | 0.63-7.06 |
| **Multivariate analysis** | | | |
| **Parameter** | **p-value** | **aOR** | **95% CI** |
| Invasive lobular BC subtype | **0.018** | **9.49** | **1.47-61.39** |
| Adjuvant breast radiation | **0.016** | **0.12** | **0.02-0.67** |

Abbreviations: BC: breast cancer, BM: brain metastasis, HER2: human epidermal growth factor receptor 2, ER: estrogen receptor, PR: progesterone receptor, HER2 subtype: HER2 positive, ER negative, PR negative, basal subtype: triple-negative, T: tumor size, N: lymph nodes, M: distant metastasis, G: grade of cancer cells, WBC: white blood cells, preop.: preoperative, MRI: Magnetic resonance imaging KPS: Karnofsky Performance Score, OR: odds ratio, CI: confidence interval, aOR: adjusted odds ratio.

**Supplementary Table E6:** Comparison of initial BC treatment of individuals with late (TI≥10 years) BM as first distant metastases without prior BC recurrence with patients developing earlier metastases and/or BC recurrence.

| **Parameter** |  | **Late metastatic patients** | **Earlier metastatic patients** | **p-value** |
| --- | --- | --- | --- | --- |
|  |  | **Nr. (%)** | **Nr. (%)** |  |
| Adjuvant breast radiation | yes | 6 (40.0%) | 56 (71.8%) | **0.033** |
|  | no | 9 (60%) | 22 (28.2%) |  |
| Breast-preserving surgery |  | 8 (53.3%) | 42 (53.8%) | 1.000 |
| Mastectomy |  | 7 (46.7%) | 36 (46.2%) |  |
| Trastuzumab | yes | 3 (20.0%) | 22 (28.2%) | 0.752 |
|  | no | 12 (80.0%) | 56 (71.8%) |  |
| HER2 RS in BC | positive | 4 (26.7%) | 25 (32.1%) | 1.000 |
|  | negative | 7 (46.7%) | 43 (55.1%) |  |
|  | n.a. | 4 (26.7%) | 10 (12.8%) |  |

Abbreviations: BC: breast cancer, BM: brain metastasis, RS: receptor status, HER2: human epidermal growth factor receptor 2, Nr.: number, TI: time interval, n.a.: not available.

**Supplementary Figure E1** ROC curve: Three survival intervals (1, 3, and 5 years) were examined to identify the most optimal TI cutoff for survival prediction. As an example, the ROC curve for 3-year survival is presented. AUC: 0.648


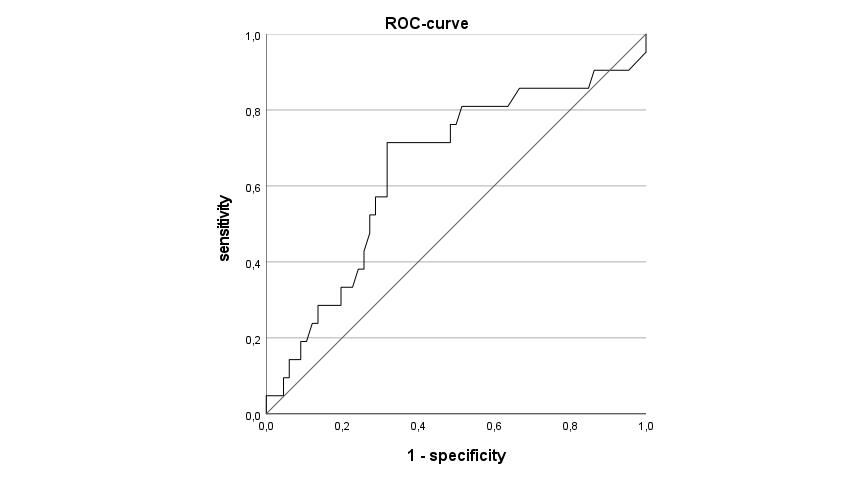


Abbreviations: TI: time interval, AUC: Areas under the curve, ROC: Receiver operating characteristic

**Supplementary Figure E2** Kaplan Meier survival plot showing the differences in TI between the individuals with LumA (=ER+PR+HER2-), LumB (=triple positive), basal (=triple negative) and HER2 (=HER2+PR-ER-) BC subtypes. The presented curves are stratified by BC subtypes with median TI of 45.0 months: LumA and LumB 54.0 months (IQR 35.00-120.00), basal and HER2 29.0 months (IQR 17.25-85.50)


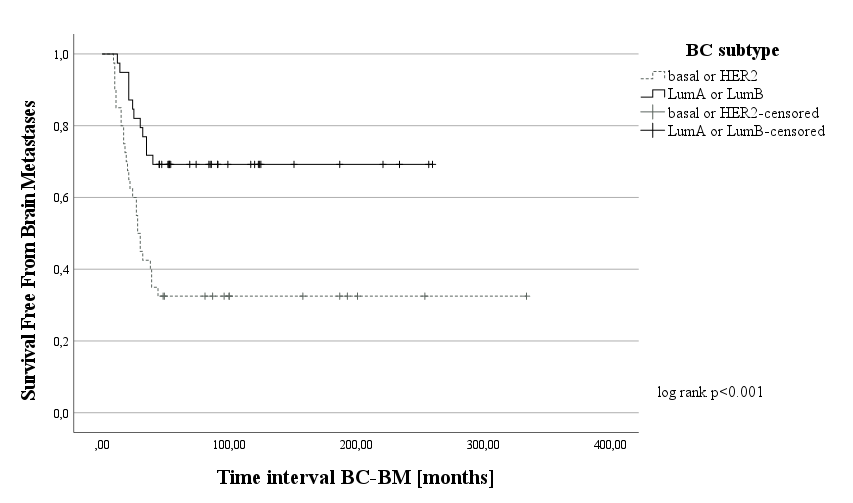


Abbreviations: BC: breast cancer, BM: brain metastasis, TI: time interval, HER2: human epidermal growth factor receptor 2, ER: estrogen receptor, PR: progesterone receptor, IQR: Interquartile ranges.

**Supplementary Figure E3** Kaplan Meier curves for illustration of TI from first BC to BM diagnosis a) stratified by histopathological subtype of BC with median TI=43.0 months (invasive ductal: 32.5 months, invasive lobular: 109.5 months), Log rank: p=0.061, b) stratified by adjuvant breast radiation with median TI=45.0 months (yes: 44.5 months, no: 45.0 months) Log rank: p=0.811,c) stratified by Trastuzumab as BC treatment with median TI=45.0 months (Trastuzumab treatment: 28.0 months, no Trastuzumab treatment: 48.0 months), Log rank: p=0.048, d) stratified by preoperative midline shift in MRI with median TI=44.5 months (midline shift: 32.0 months, not midline shift: 49.0 months), Log rank: p=0.008, e) stratified by ER receptor conversion in BM with median TI=45.0 months (identic ER status: 37.0 months, converted ER status: 74.0 months), Log rank: p=0.090, f) stratified by UICC BC stage with median TI=50.5 months (I-II: 74.0 months , III-IV: 40.0 months), Log rank: p=0.181

a) b)


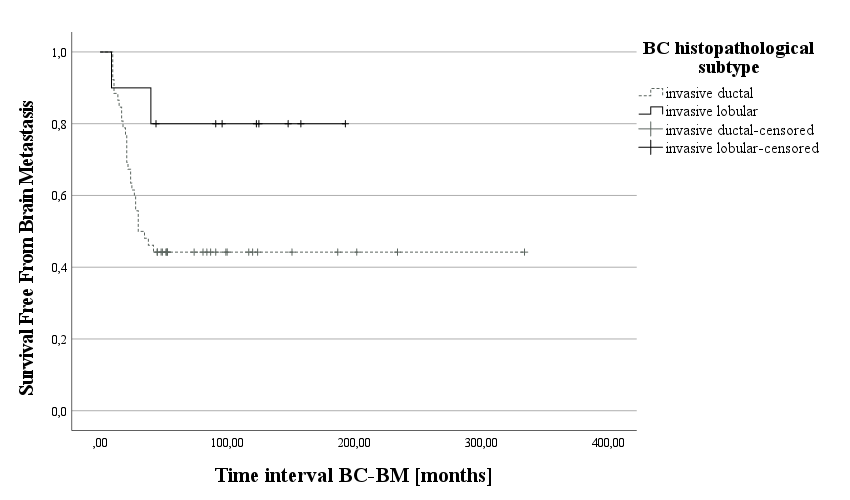

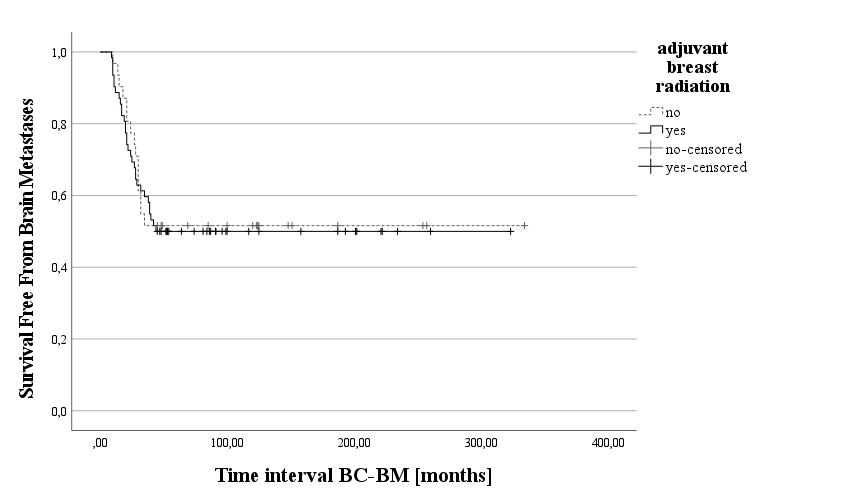


c) d)


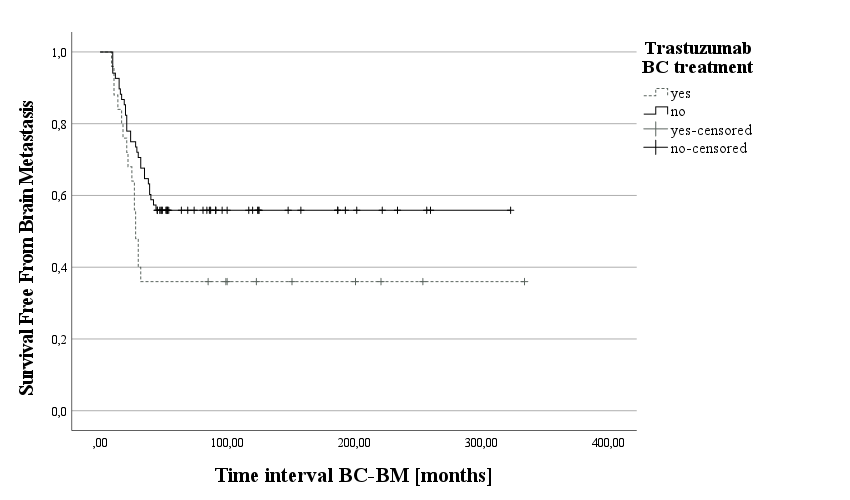

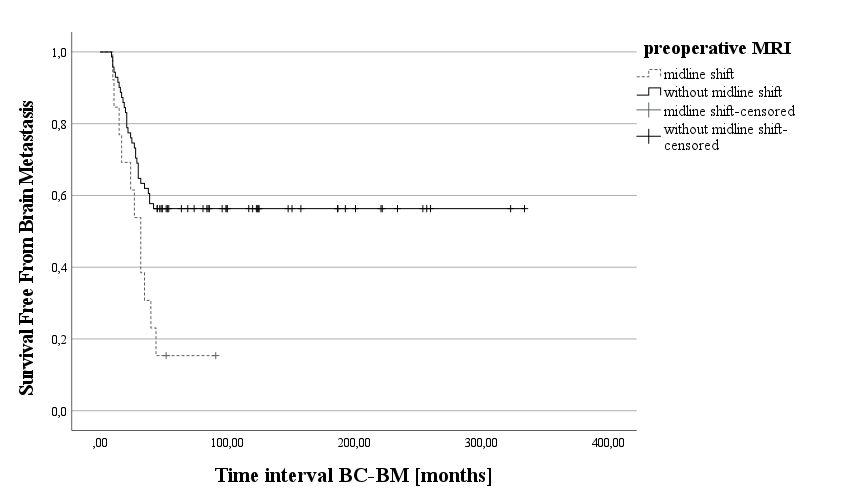


e) f)


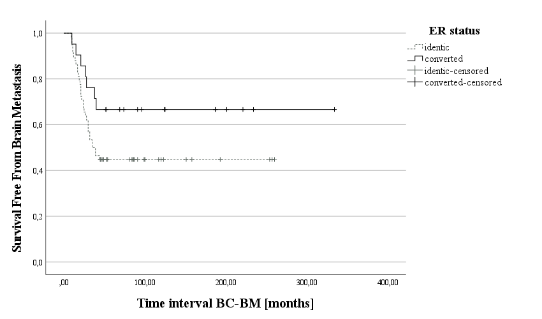

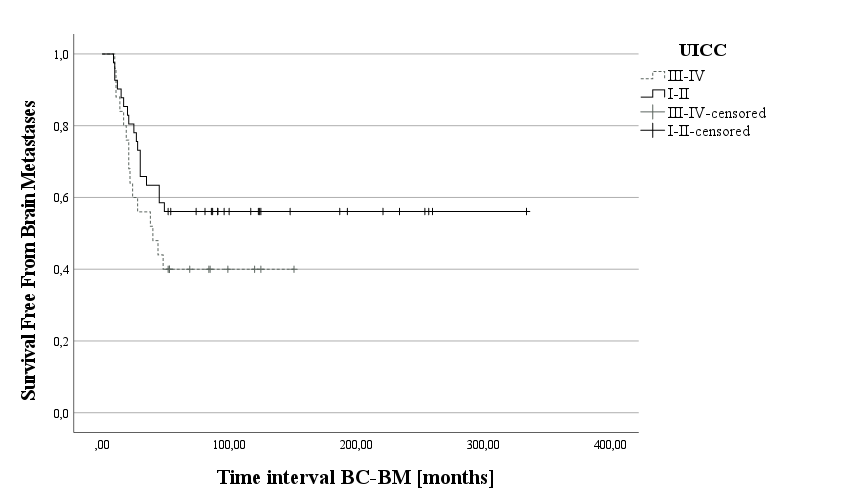


Abbreviations: BC: breast cancer, BM: brain metastasis, TI: time interval, HER2: human epidermal growth factor receptor 2, ER: estrogen receptor, MRI: Magnetic resonance imaging, UICC: Union for international cancer control
